# Level of mother’s knowledge about neonatal danger signs and associated factors in North West of Ethiopia: a community based study

**DOI:** 10.1186/s13104-015-1278-6

**Published:** 2015-07-19

**Authors:** Solomon Gedlu Nigatu, Abebaw Gebeyehu Worku, Abel Fekadu Dadi

**Affiliations:** Gondar Physiotherapy Clinic, Gondar, Ethiopia; Institute of Public Health, College of Medicine and Health Sciences, University of Gondar, Gondar, Ethiopia

**Keywords:** Neonatal danger sign, Cross sectional, Ethiopia

## Abstract

**Background:**

Neonatal danger has become a substantial problem in many developing countries like Ethiopia. More specifically, neonatal rates in Ethiopia are among the highest in the world. In this regard, health-seeking behavior of mothers for neonatal care highly relies on their knowledge about neonatal danger sign, and it has been hardly investigated. Therefore, this study was intended to determine the level of mother’s knowledge about neonatal danger signs and to identify factors associated with good mother’s knowledge.

**Methods:**

Community-based cross-sectional study was conducted from February to May 2014. A multi-stage sampling technique was used to select 603 mothers. A structured, pre-tested, and interview-administered questionnaire comprehending 13 neonatal danger signs was employed to collect the data. Data were entered into EPI-Info 3.5.2 and analyzed by SPSS version 16. Binary logistic regression model was used to identify associated factors. Odds ratio with 95% CI was computed to assess the strength and significant level of the association.

**Results:**

All mothers expected to participate in the study were interviewed. The results of the study showed that mothers who had knowledge of three or more neonatal danger signs (good knowledge) were found to be 18.2% (95% CI 15.1, 21.3%). The odds of having good knowledge was positively associated with mother’s (AOR = 3.41, 95% CI 1.37, 8.52) and father’s (AOR = 3.91, 95% CI 1.23, 12.36) higher educational achievement. Similarly, the odds of having good knowledge about neonatal danger signs was higher among Antenatal care (AOR = 2.28, 95% CI 1.05, 4.95) and Postnatal care attendant mothers (AOR = 2.08, 95% CI 1.22, 3.54). Furthermore, access to television was also associated with mothers’ good knowledge about neonatal danger signs (AOR = 3.49, 95% CI 1.30, 9.39).

**Conclusion:**

Maternal knowledge about neonatal danger signs was low. Therefore, intervention modalities that focus on increasing level of parental education, access to antenatal and postnatal care and PNC service, and advocating the use of television was pinpointed.

## Background

Globally, every year about four million babies die in the first 4 weeks of life. A similar number of babies are still born [1]. A neonatal period is only 28 days and yet accounts for 38% of all deaths in children younger than age 5 years [2]. The highest numbers of neonatal deaths are in South-Central Asian countries, and the highest rates are generally in Sub-Saharan Africa [3].

The majority of this new born deaths occur at home where a few families recognize signs of newborn illness and nearly all neonates are not taken to health facilities when they were sick [4]. The Millennium Development Goal for reducing child deaths cannot be met unless we do more to reduce neonatal deaths, especially in Africa and South Asia [5–7].

The highest rates of neonatal death (34 deaths per 1,000 live births in 2011) are still in Sub-Saharan Africa, which accounts for 38% of the global neonatal death [3]. In Ethiopia, 120,000 babies die every year in the first 4 weeks of life [8]. Similarly, a 2011 EDHS reported a 37% neonatal mortality rate and a 59% infant mortality rate [9]. Nearly 90% of deliveries in Ethiopia took place at home with low other maternal health service coverage [10, 11].

Neonates are more prone to show subtle signs of illness. Slowness or difficulty of feeding is sometimes the only signs present, and the three major causes of neonatal deaths worldwide are infections, diarrhea (36%), prematurity (28%) and birth asphyxia (23%) [2, 12, 13].

Some of repeatedly reported neonatal danger signs include not able to feed, movement only when stimulated, low or high temperature, respiratory rate over 60 breaths per minute, severe chest in drawing and history of convulsion. Recognizing the occurrence of these signs will results in high overall sensitivity and specificity to predict the need for seeking treatment of the new born [8, 14].

It is estimated that 75% of neonatal deaths could be avoided with simple low cost tools like: antibiotics for pneumonia and sepsis, sterile blades to cut the umbilical cords, and using knit caps and kangaroo care to keep babies warm [5]. This is only possible if mothers’ knowledge regarding the above neonatal danger signs is good enough to make decision to seek health service. Different tools to facilitate identification of neonatal health problems and management were introduced into the health programs in several countries like Ethiopia. Integrated Management of Newborn and Childhood Illness developed by WHO was the one which focused on assessment of neonatal danger signs and apply prompt timely treatment [14].

So far, studies in different countries reported the inconsistency of finding related to level of mothers’ knowledge and related factors about neonatal danger signs. Three and above neonatal danger signs were mentioned among 28, 13.9, 20.3 and 29% of mothers included in the study from Afghanistan [15], India [16, 17], Ghana [18], and four regions of Ethiopia respectively [19]. The repeatedly reported danger signs were: difficulty in breathing, poor sucking of breast milk, and lethargy/unconsciousness [15, 18].

Articles from Uganda, Ghana, and India reported the positive effect of birth preparedness, exposure to TV/Radio, and older age of mother to improve the knowledge of maternal key danger signs [17, 20–22]. In contrast, studies elsewhere have shown that there had been absence of relationship between age, educational status of mother, birth order, place of birth, ANC, access for skilled birth attendance, wealth, and parity [20, 23].

Generally, reducing neonatal morbidity and mortality requires immediate caregiver’s recognition of suggestive danger signs in the neonates and visiting the nearby clinic. Trends in Ethiopian society so far recognized mothers as caretakers for the majority of neonates [11, 18, 24]. Therefore, improving maternal knowledge concerning neonatal danger sign is a key entry point. However, studies on the area are limited and inconsistent.

Thus, the main aim of this study was to assess the level of maternal knowledge on neonatal danger signs and its associated factors among mothers who gave birth in the last 6 months in Gondar town, North West of Ethiopia.

## Methods

### Study setting and design

Gondar town administration is located 723 km away from North West of Addis Ababa, Ethiopia. According to the 2007 Ethiopian census report, Gondar had a total population of 206,987 and 7,878 annual live births. The town was subdivided into 12 administrative areas, and the health coverage of the city reached 74.96% in 2013. The study was conducted from February to May 2014. The study employed community based cross-sectional design including all mothers who have been living in the town for the past six consecutive years and who gave birth 6 months prior to the survey. Mentally and physically incapable women’s to provide response during data collection period were excluded.

### Study sampling

The total sample of the study was determined by using single population proportion formula by assuming 5% level of significance, 5% margin error and taking 29% proportion of good maternal knowledge on neonatal danger signs. Considering cluster effect of two and 10% non-response rate, the final sample size obtained was 575. Cluster sampling was employed to select four administrative unites, and all eligible mothers in selected unites were interviewed. Because of the cluster effect, finally 603 mothers recruited in the selected cluster were interviewed.

### Data collection and analysis

Pre-tested and interview administered questioner adopted from different literatures were employed to record mother’s knowledge about neonatal danger signs, socio-demographic, economic and obstetric related factors. Six trained first-degree health professionals conducted the data collection process. Danger signs are symptoms that complicate the lives of the neonate and happen during the first 28 days. The total number of correct spontaneous responses to 13 items with a minimum score of 0 and maximum of 13 was used to measure knowledge of women about neonatal danger signs. Accordingly, two categories were developed for neonatal danger sign. Spontaneous response is respondents naming of neonatal danger signs without giving option of the respected signs.

Women who mentioned at least three danger signs of neonate were considered as had good knowledge about neonatal danger signs and women who did not mentioned at least three danger signs of neonate were considered as had poor knowledge about neonatal danger signs [14].

The completeness and consistency of the data were checked, cleaned and double entered to Epidemiological Information (EPI-INFO) software version 3.5.1 and analyzed by Statistical Package for Social Sciences (SPSS) software version 16. Frequencies, proportions and summary statistics were used to describe the study population in relation to relevant variables and presented by using tables and graphs.

A bi variable logistic regression model was fitted to identify factors, which were significant at *p* value of less than 0.2. Those were then entered into multiple logistic regression model to handle potential confounding variables and to identify independent factors those affected mothers’ knowledge about neonatal danger signs. Odds ratio with 95% CI was used to identify significant factors. Model fitness test was conducted with Hosmer and Lemeshow goodness of fit test (Chi square = 10.337, p-value = 0.24).

### Ethical consideration

Ethical clearance was obtained from the research review ethical committee of the University of Gondar. Communication with the city and sub-city administrators was made through formal letter obtained from the University of Gondar. Having finished informing the purpose and objective of the study, the researchers obtained a written consent of the study participants with age greater than 18 years. Moreover, written consent was obtained from caretakers on behalf of those with age less than 18 years. Participants were informed that their participation was on voluntary base, and the information obtained from them was kept confidential.

## Results

All mothers expected to participate were interviewed (N = 603). The mean age of the mothers was 28 (SD ± 5.86) years. Four hundred eighty-nine (81.1%) mothers were married, and the majority of them 460 (76.3%) were Orthodox Christianity followers. One hundred forty-two (23.5%) mothers had completed grade 9 and 10. Regarding occupational status, 305 (50.6%) were housewife, 134 (22.2%) were governmental employee, and 121 (20.1%) were private employee. (Table 1)

**Table 1:** Socio-economic and obstetric characteristic of mothers of mothers in Gondar town, Northwest Ethiopia (N = 603), May 2014

Variable	Frequency	Percentage (%)
Age group		
15–24	176	29.2
25–34	330	54.1
35–44	91	15.1
45+	6	1.6
Marital status		
Single	45	7.5
Married	489	81.1
Divorced	40	6.6
Widowed	29	4.8
Religion		
Orthodox	460	76.3
Muslim	90	14.9
Protestant	44	7.3
Other	9	1.5
Educational status		
Cannot able to read and write	75	12.4
Can read and write	70	11.6
Grade 1–8	109	18.1
Grade 9–12	272	45.1
College and above	77	12.8
Occupation		
House wife	305	50.5
Government employee	121	20.1
Private employee	134	22.2
Merchant	29	4.8
Others	14	2.3
ANC attendance		
Yes	350	58
No	253	42
Frequency of visit		
<4 visits	172	49.1
≥4 visits	178	50.9
PNC attendance		
Yes	145	24
No	458	76
PNC visit		
<3	135	93.1
≥3	9	6.9
Place of delivery		
Home	194	32.2
Health institution	409	67.8
Assistance of the delivery		
Health profession	420	69.7
TBA	101	16.7
Family	82	13.6
Parity		
1	231	35.3
2–4	357	59.2
≥4	33	5.5

Among the interviewees, 350 (58%) were attended ANC for their last pregnancy, of whom, 172 (28.5%) attended less than four times. One hundred ninety-four (32.17%) mothers were gave birth at home. Of whom, 101 (55.2%) were attended by trained traditional birth attendants, and the rest, 82 (44.8%) were by their families. Four hundred-nine 409 (67.83%) mothers gave their last birth at a health institution. Mothers who lost their children were 84 (13.9%). Of whom, 37 (44%) were neonates. (Table 1)

More than 79% (79.8%) of the mothers mentioned at least one key danger sign. Two hundred-forty (39.8%) mothers’ responded high temperature, 205 (34%) mothers responded vomiting, 163 (27%) mothers responded diarrhea, and 104 (17.2%) mothers responded unable to feed as a key neonatal danger sign. One hundred-ten (18.2%) of mothers had good knowledge about neonatal danger sign (95% CI 15.1, 21.3). The most common reported source of information was health professionals (36.7%).

### Factors associated with maternal knowledge about neonatal danger signs

After controlling for socio demographic, economic, and maternal obstetric factors, mother educational status, husband educational status, attending ANC and PNC, and mothers’ access for television service were the factors that significantly affect maternal knowledge.

Mothers secondary education and above college level were three times (AOR = 3.05, 95% CI 1.43, 6.50), and more than three times (AOR = 3.41, 95% CI 1.37, 8.52) to be knowledgeable about neonatal danger signs as compared to mothers at primary education level respectively. Similarly, husbands secondary education (AOR = 3.89, 95% CI 1.29, 11.71), and above college level (AOR = 3.91, 95% CI 1.23, 12.36) were nearly four times to mention at least three neonatal danger signs as compared to husbands with primary education respectively.

Furthermore, mothers who attended ANC during the last pregnancy were two times more likely to had knowledge (AOR = 2.28, 95% CI 1.05, 4.95) about neonatal danger signs as compared to their counterpart. Similarly, mothers who had PNC follow up were two times (AOR = 2.08, 95% CI 1.22, 3.54) more likely to have good knowledge about neonatal danger signs as compared to those who did not follow. Likewise, mothers’ access to television increased their knowledge about neonatal danger signs by 3.5 times (AOR = 3.49, 95% CI 1.30, 9.39) (Table 2).

**Table 2:** Factors associated with mother’s good knowledge about neonatal danger signs, Gondar town, April 2012

Factor variables	Knowledge on neonatal danger sign		Crud OR (95% CI)	Adjusted OR (95% CI)
	Good, N	Poor, N		
Marital status				
Married	97	392		
Single	5	40	0.50 (0.19, 1.31)	
Divorce	3	37	0.33 (0.99, 1.08)	
Widowed	5	24	0.84 (0.31, 2.26)	
Mother education				
Primary education	13	241	1	1
Secondary education	68	204	6.18 (3.32, 11.51)	3.05 (1.43, 6.50)
College and above	29	48	11.2 (5.43, 23.01)	3.48 (1.37, 8.52)
Mother occupation				
Housewife	34	271	1	
Government employee	44	77	4.56 (2.72, 7.62)	
Private employee	22	112	1.57 (0.88, 2.79)	
Merchant	8	21	3.04 (1.25, 7.39)	
Others	2	12	1.33 (0.28, 6.19)	
Husband education				
Primary education	4	129	1	1
Secondary education	55	186	9.53 (3.37, 26.97)	3.89 (1.29, 11.71)
College and above	40	87	14.8 (5.12, 42.93)	3.91 (1.23, 12.36)
ANC				
No	12	241	1	1
Yes	98	252	7.81 (4.18, 14.59)	2.28 (1.05, 4.95)
PNC				
No	54	404	1	1
Yes	56	89	4.70 (3.03, 7.29)	2.08 (1.22, 3.59)
Place of delivery				
Health center	30	149	1	
Hospital	70	160	2.17 (1.34, 3.52)	
Home	10	184	0.27 (0.13, 0.57)	
Radio				
No	15	150	1	
Yes	95	343	2.77 (1.55, 4.93)	
Television				
No	11	222	1	1
Yes	99	271	7.37 (3.86, 14.09)	3.49 (1.30, 9.39)
Wealth index				
Poor	8	199	1	
Medium	31	162	4.76 (2.12, 10.64)	
Rich	71	132	13.38 (6.23, 28.70)	

## Discussion

Reduction of neonatal and infant mortality to acceptable level is impossible without good maternal knowledge regarding neonatal danger signs. This is because of the fact that, these danger signs are the entry point to provide comprehensive neonatal health care. This study presented the level and identified the contributing factors for good maternal knowledge about neonatal danger signs among mothers who gave birth in the last 6 months in Gondar town.

In this study, prevalence of mothers’ good knowledge (mothers who mentioned at least three-danger sign) was found to be 18.2%. The level of knowledge in this mothers is lower than the level reported in Afghanistan [15] and four regions of Ethiopia [19] but the level is higher than the level reported in India [17] and it is in line with neonatal danger signs knowledge level reported in Ghana [18]. The discrepancy might be because of unprompted question used for assessing the danger signs, a large sample size in the current study, and could be because of cultural differences.

This study showed that mothers’ education is an important determinant factor for mentioning of at least three neonatal danger signs. Mothers having secondary and above educational level increased the odds of their knowledge by nearly three times. The possible justification for this could be educated mothers acquire knowledge about disease and human health through their academic life. Moreover educated mothers were more likely to made decision to look for quality health service, had better access to health service information and improved perception of the danger signs. However, this finding is in part different from studies conducted elsewhere [20, 23]. The inconsistency might be due to the large sample size in the current study and the difference in study setting.

Similarly, husband education was a significant predictor of mothers’ good knowledge about neonatal danger signs. The odds of being having knowledge about neonatal danger signs was four times among mothers whose husbands achieved secondary and above educational level. This could be explained as fathers’ in culturally predominant position in a decision-making might positively affects the attitude and knowledge of the mothers.

The study also confirmed that ANC and PNC follow up practice creates a good opportunity for the mother to have good knowledge towards neonatal danger sign. Antenatal care and PNC attendant mothers were two times more likely to mention at least three neonatal danger sign as compared to their counterparts. Even though the finding is different from the study conducted in Uganda [20], there are explanations which support the current finding. The possible and the best reason for this could be mother’s exposure to ANC and PNC package, which increased the knowledge of the mother concerning the neonatal danger signs.

An increased exposure to media especially television was also increased the knowledge of mothers on neonatal danger signs. Television exposed mothers were 3.5 times more knowledgeable than their accompaniment and this is supported by a study conducted in Ghana [18]. This could be television contains a segment of airtime dedicated to teach the community about health issue of mothers and children. Using such media could also increase the memorability of the message compared to the other Medias.

Even though the community-based nature of the study improves the generalizability of the study, its cross-sectional nature affects the establishment of the cause and effect relationship between maternal knowledge regarding the danger signs and the factors that were identified. On top of that, relatively longer recall period might bias the true knowledge level of the mothers. Therefore, interpretation of the finding for decision and referencing purpose should be kept curious.

## Conclusion

Although Ethiopia has taken great initiative to empower the community to improve neonatal and infant health services at the grass root level, maternal knowledge level about neonatal danger signs, which is, a key entry point to improve neonatal health, was found to be low. This indicates that nearly 80% of mothers were more likely to delay in deciding to seek care. This could intern fires the death of neonates. Parental higher educational level achievement, attending ANC and PNC service and possessing television were the factors that were associated with good maternal knowledge towards neonatal danger sign. Thus, intervention modalities that consider the above factors might help to improve maternal knowledge regarding neonatal danger signs.

## Abbreviations

ANC: antenatal care
PNC: postnatal care
EDHS: Ethiopian Demographic and Health Survey
WHO: World Health Organization
TV: television
EPI-INFO: epidemiological information software
SPSS: statistical package for social sciences
AOR: adjusted odds ratio
CI: confidence interval
